# Molecular subtypes of triple-negative breast cancer in women of different race and ethnicity

**DOI:** 10.18632/oncotarget.26559

**Published:** 2019-01-04

**Authors:** Yuan Chun Ding, Linda Steele, Charles Warden, Sharon Wilczynski, Joanne Mortimer, Yuan Yuan, Susan L. Neuhausen

**Affiliations:** ^1^ Department of Population Sciences, Beckman Research Institute of City of Hope, Duarte, CA, USA; ^2^ Department of Cellular and Molecular Biology, Beckman Research Institute of City of Hope, Duarte, CA, USA; ^3^ Department of Pathology, City of Hope, Duarte, CA, USA; ^4^ Department of Medical Oncology, City of Hope, Duarte, CA, USA

**Keywords:** triple-negative breast cancer, molecular subtypes, hispanics, asians, african-Americans

## Abstract

Molecular subtypes of triple negative breast cancer (TNBC) are associated with variation in survival and may assist in treatment selection. However, the association of patient race or ethnicity with subtypes of TNBC and clinical outcome has not been addressed. Using nCounter Gene Expression Codesets, we classified TNBCs into subtypes: basal-like immune-activated (BLIA), basal-like immunosuppressed (BLIS), luminal androgen receptor (LAR), and mesenchymal (MES) in 48 Hispanic, 12 African-American, 21 Asian, and 34 White patients. Mean age at diagnosis was significantly associated with subtype, with the youngest mean age (50 years) in MES and the oldest mean age (64 years) in LAR (*p* < 0.0005). Subtype was significantly associated with tumor grade (*p* = 0.0012) and positive lymph nodes (*p* = 0.021), with a marginally significant association of tumor stage (*p* = 0.076). In multivariate Cox-proportional hazards modeling, BLIS was associated with worst survival and LAR with best survival. Hispanics had a significantly higher proportion of BLIS (53%, *p* = 0.03), whereas Asians had a lower proportion of BLIS (19%, *p* = 0.05) and a higher proportion of LAR (38%, *p* = 0.06) compared to the average proportion across all groups. These differences in proportions of subtype across racial and ethnic groups may explain differences in their outcomes. Determining subtypes of TNBC facilitates understanding of the heterogeneity of the TNBCs and provides a foundation for developing subtype-specific therapies and better predictors of TNBC prognosis for all races and ethnicities.

## INTRODUCTION

Women who present with triple negative breast cancer (TNBC) have worse prognoses than those with other breast cancer subtypes. Defined as breast cancer that lacks expression of estrogen receptor (ER), progesterone receptor (PR), and human epidermal growth factor receptor 2 (HER-2), TNBC is an aggressive histological subtype of breast cancer where women present with high grade, advanced disease. There are limited treatment options and very poor prognosis following progression [1]. Compared to other types of breast cancer, women with TNBC develop recurrent disease early and often to visceral sites. TNBC is sensitive to chemotherapy but responses are short-lived and median survival for those with metastatic disease is only 12 months [2].

TNBC accounts for 10–25% of all invasive breast cancers depending on race and ethnicity. In a 2010 study of 57,483 breast cancer patients from 17 population-based registries participating in the Surveillance, Epidemiology, and End Results (SEER) program, African-American women were twice as likely and Hispanic women were 30% more likely to be diagnosed with TNBC than non-Hispanic Caucasians (hereafter referred to as Whites) [3]. This was consistent with a report from the California Cancer Registry found that women with TNBC were significantly more likely to be African-American or Hispanic and diagnosed under the age of 40 years [4]. The higher incidence of TNBC in women of African ancestry [5] may explain the worse prognosis and higher mortality from breast cancer among African-American [4, 6, 7] compared to White women.

Molecular subtypes of TNBC based on RNA profiling have been shown to be prognostic and predictive of pathological response to neoadjuvant therapy [8–10]. These molecular classifications present an opportunity to improve therapies and therapeutic choices. What has not yet been determined is whether the TNBC molecular subtypes associate with disparities in clinical outcome across race and ethnicity. To date, compared to Whites, few non-White women have been included in studies defining TNBC subtypes, even though both Hispanics and African Americans have a higher proportion of TNBC than Whites [5]. Even within the Cancer Genome Atlas (TCGA) breast cancer set of more than 1000 women, there are few samples from non-White women with TNBC. In this study, we characterized the TNBC subtypes of 48 Hispanic, 12 African-American, 21 East Asian, and 34 White patients and determined the association of these subtypes with clinical outcomes.

## RESULTS

### Patient characteristics

As shown in Table 1, there were no statistically significant differences in characteristics across race and ethnicity. The mean age of patients was 56 years (range 27 to 91). There were 34 Whites, 48 Hispanics, 12 African Americans, and 21 Asians. The majority of women (68%) presented with self-palpated breast mass (Table 1). Of the women, 38% of Hispanics, 58% of African Americans, 32% of Whites, and 38% of Asians reported a family history of breast cancer in first- and/or second-degree relatives. There was a marginally statistically significant association (*p* = 0.06) for positive lymph nodes where 43% of Asians had positive lymph nodes compared to an average of 23% across all 115 TNBC cases.

**Table 1:** Characteristics of the 115 participants by race and ethnicity

	White	Hispanic	African American	Asian	Total	*p*-value
Number	34	48	12	21	115	
Mean age at diagnosis	59.7	54.7	55.8	54	56.2	0.2893
(median; range)	(60; 35–91)	(53; 27–77)	(50; 37–85)	(52; 41–76)	(54; 27–91)	
Family history^*^	Number (%)					
Yes	11 (32.3)	18 (37.5)	7 (58.3)	8 (38.1)	44 (38.0)	0.4811
No	23 (67.7)	30 (62.5)	5 (41.7)	13 (61.9)	71 (62.0)	
How first detected	Number (%)					
Lump felt	23 (67.7)	34 (70.8)	7 (58.3)	13 (61.9)	78 (68.0)	0.6304
Mammogram	8 (23.4)	12 (25.0)	5 (41.7)	5 (23.8)	30 (26.0)	
Other	3 (5.9)	2 (4.2)	0	3 (14.3)	7 (6.0)	
Tumor grade	Number (%)					
Grade 2	3 (8.8)	5 (10.4)	3 (25.0)	6 (28.6)	17 (14.8)	0.1383
Grade 3	28 (82.4)	43 (89.6)	9 (75.0)	15 (71.4)	95 (82.6)	
Missing	3 (8.8)	0	0	0	3 (2.6)	
Tumor stage	Number (%)					
1	10 (29.4)	15 (31.3)	4 (33.3)	4 (19.1)	33 (28.7)	0.8358
2	21 (61.8)	28 (58.3)	6 (50.0)	13 (61.8)	68 (59.1)	
3	3 (8.8)	5 (10.3)	2 (16.7)	4 (19.1)	14 (12.2)	
Tumor size						
T1	10 (29.4)	16 (33.3)	6 (50.0)	8 (38.1)	40 (34.8)	0.4917
T2	20 (58.8)	29 (60.4)	4 (33.3)	10 (47.6)	63 (54.8)	
T3	4 (11.8)	2 (4.2)	2 (16.7)	2 (9.5)	10 (8.7)	
T4	0	1 (2.1)	0	1 (4.8)	2 (1.7)	
Positive lymph nodes	Number (%)					
Yes	7 (20.6)	7 (14.6)	3 (25.0)	9 (42.9)	26 (22.6)	0.0583
No	26 (76.5)	41 (85.4)	8 (66.7)	11 (52.4)	86 (74.8)	
Missing	1 (2.9)	0	1 (8.3)	1 (4.7)	3 (2.6)	

^*^Family history of breast cancer in first- or second-degree relative.

### Classification of subtypes of TNBC

Burstein *et al.* [11] defined four stable TNBC subtypes, basal-like immune-activated (BLIA), basal-like immunosuppressed (BLIS), luminal androgen receptor (LAR), and mesenchymal (MES), using the non-negative matrix factorization (NMF) method and determined 80-gene subtype centroid signatures to classify the four NMF-defined TNBC subtypes; we downloaded those data (GSE76124) and replicated the subtype assignment using the Burstein 80-gene centroid signatures. In addition to the 80 genes, we measured expression of 11 genes that identified subtypes of TNBC that were prognostic in a methylome study [12]. Using prediction analysis of microarray (PAM) to optimize the set of genes that could best discriminate the subtypes, we found that the optimal set comprised 77 genes (see Supplementary Table 1 for gene list). Based on a comparison of inconsistencies in subtype calls between the NMF-defined subtypes and subtypes assigned using Burstein's 80-gene centroid signature or comparing the NMF-defined subtypes to subtypes assigned using our 77-gene centroid signatures, we found that the 77-gene signatures had improved accuracy of subtype prediction; there were fewer inconsistencies for the 84 training sample set (inconsistencies of 5 in the 80-gene signatures versus 2 in the 77-gene signatures) and the 114 validation sample set (inconsistencies of 23 in the 80-gene signatures versus 19 in the 77-gene signatures). Therefore, the analyses results presented below use this 77-gene signatures. For the 115 samples, we identified the four signatures of BLIA, BLIS, LAR, and MES (Table 2). MES was rare, only being identified in four samples (3.5%) compared to 18% by Burstein *et al.* [11]. Two cases were unclassified because there was poor assignment to any subtype (both had no centroid correlations > 0.55); these two samples were excluded from further analyses.

**Table 2:** Clinical features of 113 breast cancer cases by subtype

	BLIA	BLIS	LAR	MES	*P*-value
Number	38	46	25	4	
Mean Age at diagnosis	51	55	64	50	0.0005
(median; range)	49; 36–85	56; 27–77	63; 46–91	46; 35–76	
Family history^*^	Number (%)				
Yes	18 (47.4)	17 (37.0)	8 (32.0)	0	0.2642
No	20 (52.6)	29 (63.0)	14 (68.0)	4 (100.0)	
Race and ethnicity	Number (%)				
White	10 (30.3)	12 (36.4)	9 (27.3)	2 (6.1)	0.1811
Hispanic	14 (29.8)	25 (53.2)	6 (12.8)	2 (4.3)	
African American	5 (41.7)	5 (41.7)	2 (16.7)	0	
Asian	9 (42.9)	4 (19.1)	8 (38.1)	0	
Tumor grade	Number (% of those graded)				
Grade 2	2 (5.3)	5 (10.9)	10 (43.5)	0	0.0012
Grade 3	36 (94.7)	41 (89.1)	13 (56.5)	4 (100.0)	
Missing	0	0	2	0	
Tumor stage	Number (%)				
1	13 (34.2)	12 (26.1)	6 (24.0)	2 (50.0)	0.0761
2	22 (57.9)	31 (67.4)	11 (44.0)	2 (50.0)	
3	3 (7.9)	3 (6.2)	8 (32.0)	0	
Positive lymph nodes	Number (% of those examined)				
Yes	10 (27.8)	5 (10.9)	10 (41.7)	1 (25.0)	0.0211
No	26 (72.2)	41 (89.1)	14 (58.3)	3 (75.0)	
Missing	2	0	1	0	

^*^Family history of breast cancer in first- or second-degree relatives.

### Molecular subtypes of TNBCs and association with clinical features and race and ethnicity

Clinical features for the BLIA, BLIS, MES, and LAR subtypes are shown in Table 2. Mean age at diagnosis was significantly associated with subtype (*p* = 0.0005), with the MES group having the youngest mean age (50 years) and the LAR group having the oldest mean age (64 years) at diagnosis. For tumor characteristics, tumor grade (*p* = 0.0012) and positive lymph nodes (*p* = 0.021) were significantly associated with subtype; there was a marginally significant association of tumor stage and subtype (*p* = 0.076). There was no association of family history or race and ethnicity with the overall distribution of the four subtypes.

### Associations of molecular subtypes with recurrence-free and overall survival

**Figure 1:**
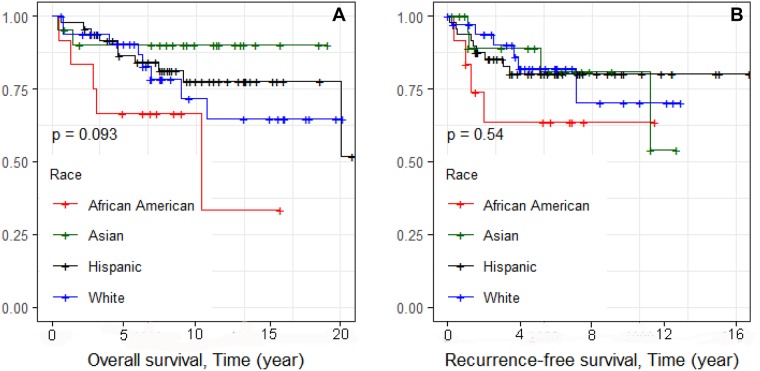
Kaplan–Meier analyses of overall and recurrence-free survival and race/ethnicity for 115 TNBC cases (Figure 1A and 1B, respectively)

**Figure 2:**
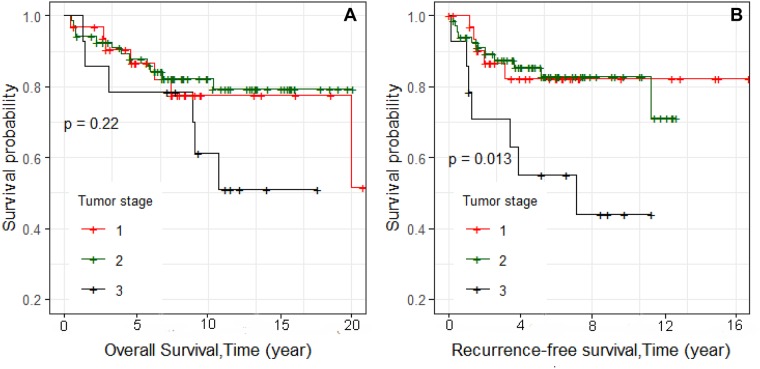
Kaplan–Meier analyses of overall and recurrence-free survival and stage for 115 TNBC cases (Figure 2A and 2B, respectively)

**Figure 3:**
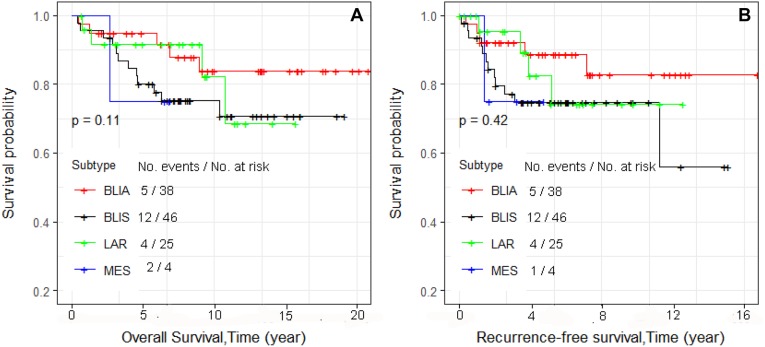
Kaplan–Meier analyses of overall survival (**A**) and recurrence-free survival (**B**) and four subtypes for 113 TNBC patients. Number of patient at risk and cumulative number of events over a time period were summarized in each plot.

**Figure 4:**
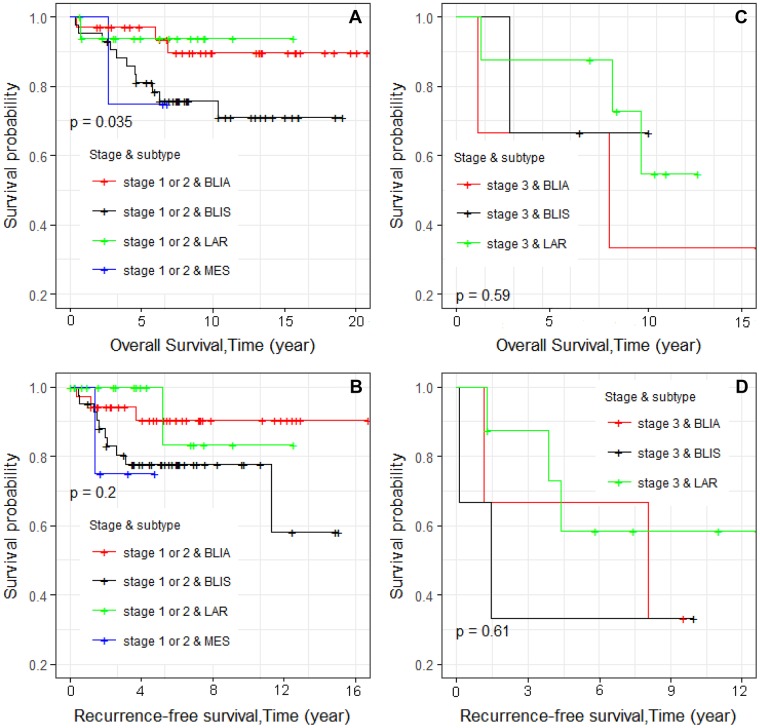
Kaplan–Meier analyses of overall survival and recurrence-free survival and tumor subtype for 113 TNBC patients stratified by tumor stages 1 and 2 and tumor stage 3 (**A**–**D**, respectively).

Kaplan–Meier survival curves were constructed. We first examined overall survival and recurrence-free survival for each race and ethnic group (Figure 1A and 1B, respectively). Consistent with reports of others, African Americans had worse overall survival, and Asians had the best survival [13]. We also examined differences in overall survival and recurrence-free survival by stage (Figure 2A and 2B, respectively) and found significant differences in recurrence-free survival with Stage 1 and 2 having equivalent survival curves and Stage 3 having worse survival (*p* = 0.013; Figure 2B). In the analyses of the subtypes, there were no significant differences in either overall survival or recurrence-free survival, although the BLIA subtype had the best survival of the subtypes (Figure 3A and 3B, respectively). Because there were significant differences in stage by subtype (Table 2) and recurrence-free survival by stage (Figure 2B), we also conducted the analyses of subtype with stage 1 and 2 combined and stage 3 separately (Figure 4A–4D, respectively). There was a significant difference in overall survival by subtype (Log-rank test, *p* = 0.035) among Stage 1/2 cancers (Figure 3A) but no significant differences for recurrence-free survival in Stage 1/2 or for overall or recurrence-free survival for Stage 3 cancers although power was limited.

Cox-proportional hazards modeling was used to assess differences in subtypes for recurrence-free survival and overall survival with the reference being either BLIA subtype or BLIS subtype. Multivariate models were adjusted for age at breast cancer diagnosis, tumor stage, and race and ethnicity. As shown in Table 3, individuals with BLIS had significantly worse (HR = 11.6, *p* = 0.002) and marginally significantly worse (HR = 2.95, *p* = 0.08) recurrence-free survival than those with LAR and BLIA subtypes, respectively. For overall survival, LAR had significantly better survival as compared to both BLIA and BLIS subtypes (HR = 0.192, *p* = 0.049, and HR = 0.073, *p* = 0.001, respectively). In order to assess whether these differences were associated with race or ethnicity, we examined the distribution of these individual subtypes. Interestingly, for LAR, the percentages varied from 12.8% in Hispanics to 38.1% in Asians; for BLIS, percentages ranged from 19.1% in Asians to 53.2% in Hispanics (Table 2). Logistic regression models comparing the proportion of the BLIS and the LAR subtypes in each racial and ethnic group to the average proportion of the subtype across all four groups were used to test whether the BLIS and LAR subtypes had the same proportions in each group; Asians were less likely to have the BLIS subtype (*p* = 0.05) and more likely to have the LAR subtype (*p* = 0.06), whereas Hispanics were more likely to have the BLIS subtype (*p* = 0.03) and less likely to have the LAR subtype (*p* = 0.10; Table 4).

**Table 3:** Cox-proportional hazard models of association of molecular subtypes with recurrence-free survival and overall survival

Survival	Comparison	Adjusted HR^*^	95% CI for HR	*p* value
Recurrence-free survival	**BLIA**	1.000		
	BLIS	2.951	0.871–9.990	0.082
	LAR	0.253	0.049–1.310	0.101
	MES	6.006	0.542–66.550	0.144
	**BLIS**	1.000		
	LAR	0.086	0.017–0.420	0.002
	MES	2.036	0.221–18.740	0.531
Overall survival	**BLIA**	1.000		
	BLIS	2.611	0.788–8.660	0.115
	LAR	0.192	0.037–0.990	0.049
	MES	4.668	0.550–39.910	0.159
	**BLIS**	1.000		
	LAR	0.073	0.015–0.354	0.001
	MES	1.787	0.257–12.426	0.557

^*^HR, hazard ratio; CI, confidence interval. Cox proportional hazard regression models were used to estimate association between a subtype and overall and recurrence-free survival, including age at diagnosis, race/ethnicity, and tumor stage as covariates. Smoothed plots of weighted Schoenfeld residuals were used to test the assumption of proportionality.

**Table 4:** Association of specific subtype with race and ethnicity

	Subtype comparisons, count (%)							
Race and ethnicity	Yes-LAR	No-LAR	*p* value	coefficient	Yes-BLIS	No-BLIS	*p* value	coefficient
White	9 (27.3)	24 (72.7)	0.49	0.27	12 (36.4)	21 (63.6)	0.99	−0.01
Hispanic	6 (12.8)	41 (87.2)	0.10	−0.67	25 (53.2)	22 (46.8)	0.03	0.68
African American	2 (16.7)	10 (83.3)	0.55	−0.36	5 (41.7)	7 (58.3)	0.65	0.22
Asian	8 (38.1)	13 (61.9)	0.06	0.76	4 (19.1)	17 (80.9)	0.05	−0.89
Total	25 (22.1)	88 (77.9)			46 (40.7)	67 (59.3)		

The binary logistic regression model was used to assess relationship between subtype-LAR or BLIS (yes or no, response variable) and race and ethnicity (explanatory variable); the coefficient *p*-values is based on the Wald test.

## DISCUSSION

This study investigated differences in TNBC subtypes by race and ethnicity. This is particularly important given that Hispanics and Asians are fast-growing populations in the United States; however, they have been largely under-represented in genomic studies of cancer. Even within TCGA breast cancer set of more than 1000 women, there are few samples from non-White women, and particularly from those with TNBC; in total, there are 8 Asians, 4 Hispanics, and 33 African Americans with TNBC. The lack of knowledge of biological differences underlying disparities in clinical outcomes needs to be addressed.

The molecular biology of TNBC is heterogeneous. In recent studies using microarray expression data, subtypes of TNBC were identified and associated with differences in treatment response and prognosis. The initial study defined six subtypes: basal-like 1 (BL1), basal-like 2 (BL2), immunomodulatory (IM), mesenchymal (M), mesenchymal stem-like (MSL), and luminal androgen receptor (LAR) subtype [9, 10]. In a follow-up study, the IM and MSL subtypes were removed after discovering that they originated from infiltrating lymphocytes and tumor-associated stromal cells, respectively, and thus were not true TNBC tumor subtypes [14]. In a more recent study, Burstein *et al*. [11] defined four subtypes of BLIA, BLIS, MES, and LAR [11]. In a comparison of the distribution of TNBC subtypes between the Lehman and Burstein signatures using the same set of samples, the MES signature of Burstein almost completely overlapped with the IM and MSL subtypes of Lehmann *et al*. [15]; we found the same result in our comparison of 114 samples (GSE76124) where the MES subtype consisted of 31% IM and 62% MSL subtype. Because the MES largely reflects the mixture of stromal or immune cells with epithelial cells, the MES subtype may depend on how carefully epithelial cells were selected from FFPE blocks. In this study, a much lower percentage of MES subtype was observed (3.5% versus 17.8% in Burstein *et al*. [11]), possibly because of the more stringent isolation criteria used in our study.

The comparative analyses of LAR of the Lehman and Burstein signatures had significant overlap. We also found that correlation coefficients for LAR subtype were high, and there was clear definition of the subtype with the signature (Supplementary Figure 1). In contrast to the LAR signature, the BLIA and BLIS signatures were less distinct (Supplementary Figure 1). If we define a mixed subtype as one where the measured difference of the top-two Spearman correlation values in a sample is less than 0.1 [16], then 29 of 115 (25%) samples would be classified as mixed BLIA-BLIS subtypes, and another 9 of 115 (8%) samples would have other mixed subtypes (see Supplementary Table 2). There were fewer mixed subtypes among the Asian cases compared to the other groups as well as a significantly greater correlation difference between the top two subtypes (*p* < 0.05) (Supplementary Table 2 and Supplementary Figure 2). The data of Burstein *et al*. and of Lehman *et al*. were similar to our results for mixed subtypes in that 27 of the 114 Burstein validation samples (23.7%) and 76 of the 243 Lehman validation samples (31.3%) were a mix of two subtypes based on this cut-off. This suggests that tumors are heterogeneous and contain multiple subtypes and/or that the subtype signatures may require further refinement. Interestingly, we found that when including the BLIS-BLIA mixed subtype in Kaplan–Meier analyses, this mixed group subtype fell between the BLIA and BLIS subtypes for recurrence-free survival and overall survival (see Supplementary Figure 3).

The recent development of molecular classifiers of TNBC presents a real opportunity to improve therapies and therapeutic choices. Potential subtype-specific therapeutic targets previously were identified based on the unique gene expression profiles [11] of their 80-gene signature. In order to potentially improve the signature, we added an additional 11 genes from a methylome study which classified TNBCs into prognostic clusters, and used NanoString assays to measure expression. This technology is robust and accurate for RNA from FFPE samples [17, 18]. Because it is hybridization-based, it does not require reverse transcription of mRNA and cDNA amplification thus reducing amplification bias; and because it is focused on a small number of genes rather than an expression array, it is more cost effective.

This study was designed to study the association of molecular subtypes of TNBCs and disparities in survival across races and ethnicities. We found that TNBC samples from Hispanic and Asian women had significantly different proportions of BLIS and LAR compared to the average proportion of corresponding subtype across all four groups. These differences in the distributions of TNBC subtypes may reflect true biologic/genetic differences by race and ethnicity. There may be better signatures to define subtypes in different racial and ethnic populations as the signatures were originally developed using TNBC samples from primarily White women. We will need to perform global RNA-seq in the future to identify if there are additional biomarkers that may separate Hispanic and Asian TNBC subtypes. Another possibility is that although our sample size is reasonable, it is divided among 4 groups of different race and ethnicity. This may make group sizes too small to have adequate power to detect all the differences. That appears to be the case for the African-American TNBC cases, which had a genetic signature more similar to the Hispanic cases, but the sample size did not have sufficient power to discern statistical differences.

This study is one of the first to conduct molecular subtyping of TNBC comparing Whites, Hispanics, Asians, and African Americans; it fills a gap of knowledge in how molecular subtypes of TNBC may be used to improve tumor-biology-driven prognosis of TNBC in these underserved and understudied populations. In multivariate analysis accounting for age and stage at diagnosis and race/ethnicity, our results show that TNBC subtype BLIS is associated with worse survival and LAR with the best survival (Table 3). This result was consistent with a study comparing AR+/epidermal growth factor (EGFR) – tumors (the LAR subtype), in which they reported that LAR had the best prognosis compared to the basal TNBC subtype (defined as AR−/EGFR+) [19]. In univariate Kaplan–Meier analyses, our results were consistent with those reported by Burstein *et al*. [11]; although not statistically significant, we found that BLIS had the worst survival and BLIA the best survival compared to the other subtypes. Our results differ from those reported by Lehmann *et al*. [14] where they observed non-significantly worse survival for LAR. There likely are several reasons for this inconsistency: 1) these are chance differences as their findings were not statistically significant; 2) we conducted multivariate analyses adjusting for age at diagnosis and stage of the cancer; both factors that were significantly associated with subtype (Table 2); 3) LAR is the only subtype that overlaps between the Burstein *et al*. [11] and Lehmann *et al*. subtypes [14]; 4) we excluded women who were treated with neoadjuvant therapy – pathological complete response for LAR is worse than for BL1 which would reflect in Kaplan–Meier analyses. The Asian women in the study had significantly lower numbers of BLIS and higher numbers of LAR, and they showed the best overall survival which is consistent with what has been previously reported for survival [13]. From a public health perspective, the sub-typing of TNBCs may facilitate understanding of the heterogeneity of the TNBCs and provide a foundation for developing subtype-specific therapies and better predictors of TNBC prognosis for all races and ethnicities.

## MATERIALS AND METHODS

### Identification and selection of patients

Paraffin blocks from formalin-fixed primary breast cancer tissue specimens were obtained from the City of Hope Biorepository through an Institutional Review Board approved protocol. Patients were selected on the basis of self-reported ethnicity and race, TNBC diagnosis was from January 2000 through July 2016, and tumor tissue availability. Tissue samples were procured from 115 patients with TNBCs who had not had neo-adjuvant therapy. Our primary focus was Hispanic Caucasian women primarily from Mexico, referred to as Hispanic. We then selected Asians (East Asians, primarily Han Chinese) and African Americans and then Whites (non-Hispanic Caucasians). Data on family history of breast cancer, age, pathology stage, receptor status, chemotherapy regimens, time to recurrence, and cause and date of death (if occurred) were captured through chart review.

### RNA preparation

A surgical pathologist identified foci with greater than 80% tumor on hematoxylin and eosin stained slides from formalin-fixed paraffin-embedded (FFPE) tissue. For RNA extraction, the corresponding areas were microdissected from 10-micron unstained sections. The specific area(s) from the unstained sections were then dissected for extraction of RNA. RecoverAll™ Total Nucleic Acid Isolation kit (Ambion) was used to extract total RNA following the manufacturer's instructions. RNA was quantified by fluorometry using Quant-iT RNA BR Assay Kit. Quality was assessed using the 2100 Bioanalyzer (Agilent Technologies).

### Gene expression assays

For each FFPE sample, 250 ng of extracted RNA was isolated for analysis by nCounter Gene Expression Codesets (NanoString Technologies). For gene expression profiling, we measured expression of 99 genes; this set included the 80 genes in the expression signature in Burstein *et al*. [11], 12 genes from a methylome study that identified prognostic clusters (1 overlaps with Burstein gene signature set) [12], and 8 housekeeping genes (*ACTB, GUSB, MRPL19, PSMC4, PUM1, RPLP0, SF3A1,* and *TFRC*) for normalization from PAM50 (Prosigna). In addition to the 115 patient samples described above, an additional 5 TNBC samples with accompanying Affymetrix expression array data were tested as known control subtypes to ensure that this first use of NanoString technology to define subtypes faithfully recapitulated the Affymetrix expression signatures. The COH Molecular Diagnostic Lab processed the RNA samples and produced files with raw counts for each gene in each sample.

### Data analysis

nCounter raw data were background assessed and quality checked using nCounter internal negative and positive controls by following the data analysis workflow from NanoString Technologies. Using nSolver Analysis Software, the absolute transcript abundance was determined for each sample by further normalizing expression with the housekeeping genes listed above. Log2 transformation of normalized gene expression data was used for TNBC subtype classification. We used the gene expression data (GSE76124) from the Burstein *et al*. [11] dataset, which included 84 samples in the training set and 114 samples in the validation set. Because we added 11 genes to the gene signature of Burstein *et al*. [11] (described above under Gene Expression assays), PAM method [20, 21] was used to select the optimal subset of genes to classify the four TNBC subtypes BLIA, BLIS, LAR, and MES. From this analysis, a gene set was selected, and quantile expression centroids of the four subtypes of TNBC were calculated from the 84 training samples.

To account for batch effects between the Burstein training data set [11] and our test data, the addon batch adjustment technique [22, 23] was used to adjust our test data to the Burstein training data; the prediction rules generated from the training data were used to predict subtypes in our data. Principal component analysis (PCA) was used to assess the effect of the addon batch adjustment (see Supplementary Figure 4). Before the batch adjustment, PCA results showed spatial separation of the training data from our test data (Supplementary Figure 4A); after batch adjustment, the two data sets were spatially aligned (Supplementary Figure 4B). Then one of four TNBC subtypes was assigned to each of our 120 samples (5 control samples with Affymetrix gene expression data and 115 test samples) based on the strongest Spearman correlation between sample expression and the 77-gene subtype centroid signatures (see Supplementary Table 2). The subtype was assigned based on the highest Spearman correlation coefficient regardless of the difference between the top two Spearman correlation values. For the five control samples, two were assigned to the BLIA subtype, two to the BLIS subtype, and one to the MES subtype, all consistent with the subtypes assigned using Affymetrix gene expression data.

### Statistical analyses

Patient and disease characteristics included: a) age, stage at diagnosis, race or ethnicity, and family history of breast cancer; b) overall survival defined as time from the date of surgery to death from any cause; and c) recurrence-free survival defined as time from date of surgery to breast cancer recurrence. Fisher's exact test was used to test the association between subtypes and categorical clinical variables including tumor stage, race, tumor grade, positive lymph node, and family history of breast cancer (yes/no). Binary logistic regression analysis was used to assess the direction and strength of association between a specific subtype (response variable; yes or no) and race or ethnicity (explanatory variable).

For recurrence-free survival, time was censored at death if cause of death was not from breast cancer or at last contact if the patient was still alive at last contact date. For overall survival, time was censored at last contact date. For univariate analysis, overall and recurrence-free survival curves were generated by Kaplan–Meier method, and a log-rank test was employed to assess survival difference. For multivariate analysis, Cox regression models were used to estimate association between a subtype and overall and recurrence-free survival, including age at diagnosis, race or ethnicity, and tumor stage as covariates. Smoothed plots of weighted Schoenfeld residuals generated by the cox.zph function of survival package in R were used to test the assumption of proportionality for all predictors in the Cox model [24]; results are shown in Supplementary Figure 5.

## SUPPLEMENTARY MATERIALS FIGURES AND TABLES







## Abbreviations

TNBC: triple negative breast cancer
HR: hazard ratio
CI: confidence interval
BLIA: basal-like immune activated
BLIS: basal-like immunosuppressed
LAR: luminal androgen receptor
MES: mesenchymal
TCGA: The Cancer Genome Atlas
NMF: non-negative matrix factorization.
