# Supplementary material for: Molecular subtypes of triple-negative breast cancer in women of different race and ethnicity
Source: Oncotarget. 2019 Jan 4;10(2):198–208. doi: 10.18632/oncotarget.26559 (PMC6349443; doi:10.18632/oncotarget.26559)
Supplement: Supplementary file 1 [file oncotarget-10-198-s001.pdf]

Molecular subtypes of triple-negative breast cancer in women of different race and ethnicity

SUPPLEMENTARY MATERIALS

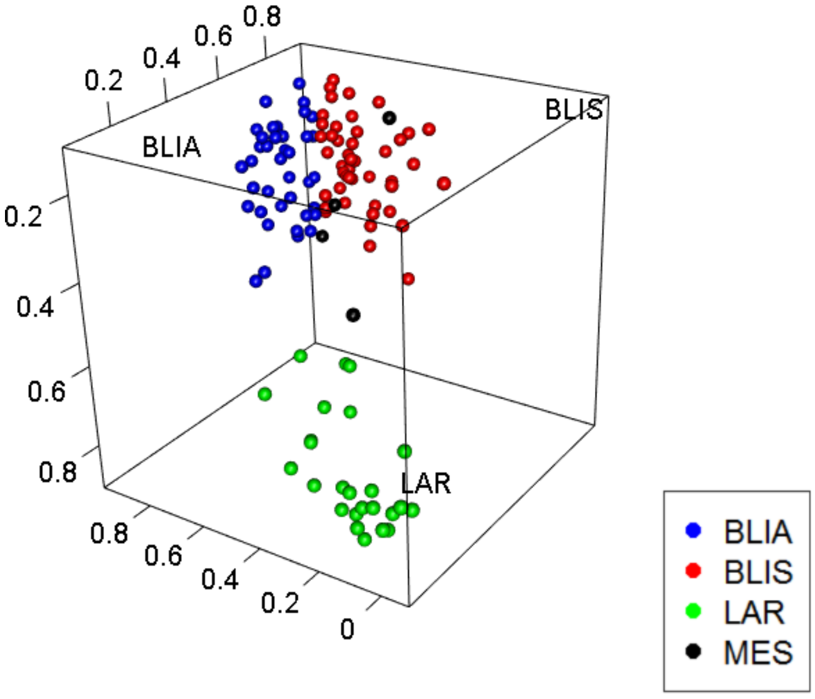

Supplementary Figure 1: Three-dimensional display of subtype distribution in 113 samples using Spearman correlation coefficients for BLIS, BLIA, LAR subtypes as x, y, and z axis values, respectively.

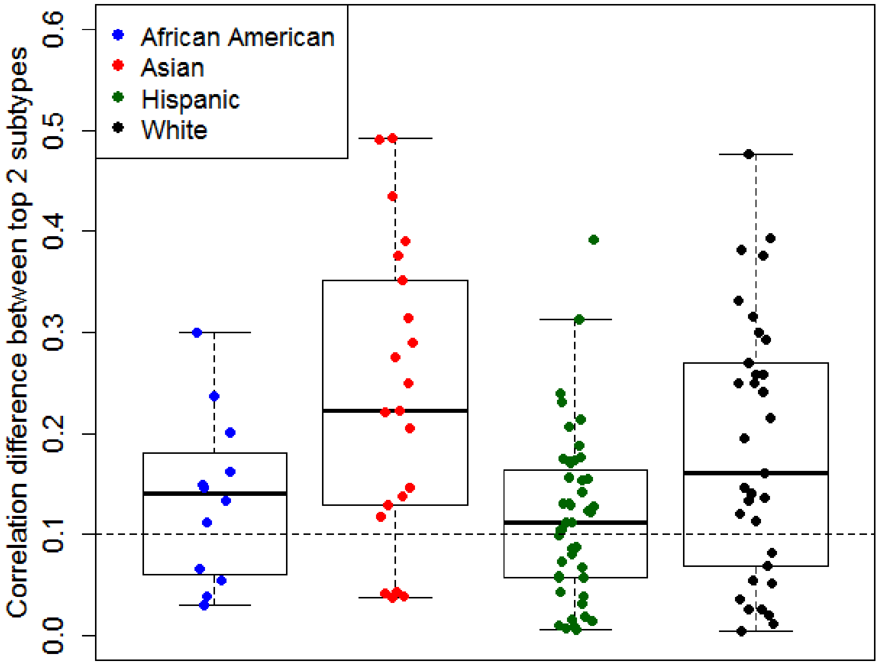

Supplementary Figure 2: Distribution of correlation difference between top-two subtypes in difference population groups. A sample is allowed to be associated with multiple subtypes if correlation difference between top-two subtypes in the sample is less than 0.1 (dash line).

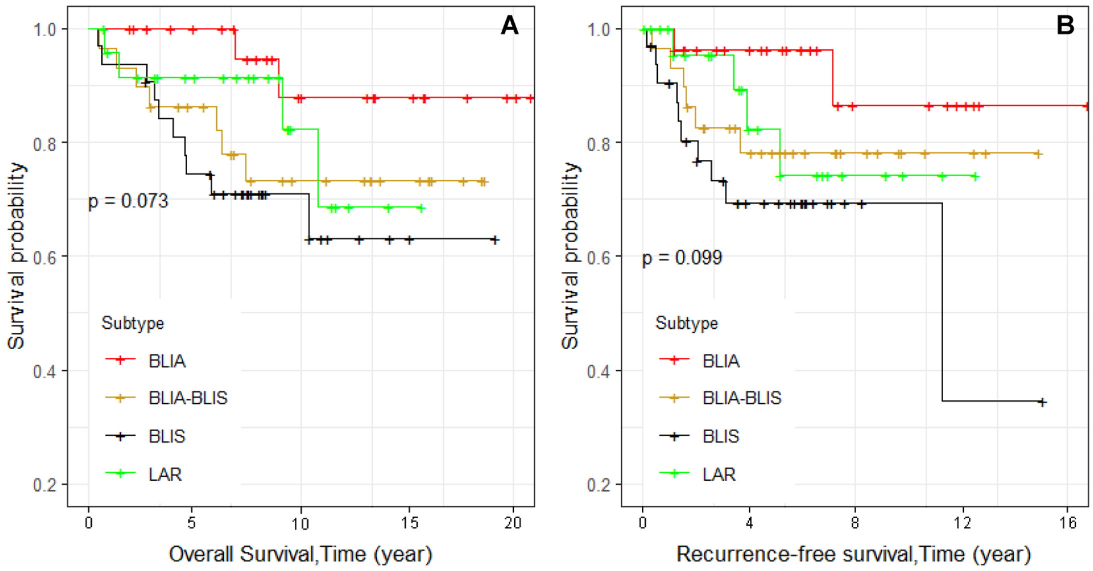

Supplementary Figure 3: Kaplan–Meier analyses of overall survival (**A**) and disease-free survival (**B**) for 112 TNBC patients stratified by subtypes. The subtype for each sample was assigned based on the highest Spearman correlation coefficient. A sample was considered to carry a mixture of subtypes if the difference of the top-two Spearman correlation values in a sample was less than 0.1. Because there was only one sample predominantly carrying the MES subtype, MES was not included in these analyses.

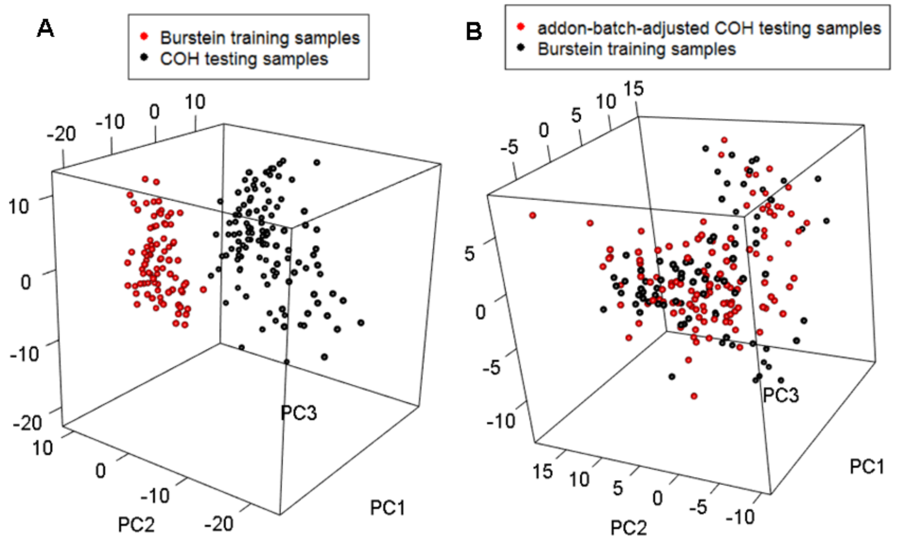

Supplementary Figure 4: Using principal component analysis, distributions of subtypes in the Burstein *et al.* training set and our data set are shown: (**A**) before adjusting for batch effects, the two data sets are spatially different; (**B**) after performing addon-batch adjustment, the batch effect is no longer observed as the samples from the two datasets are now spatially aligned.

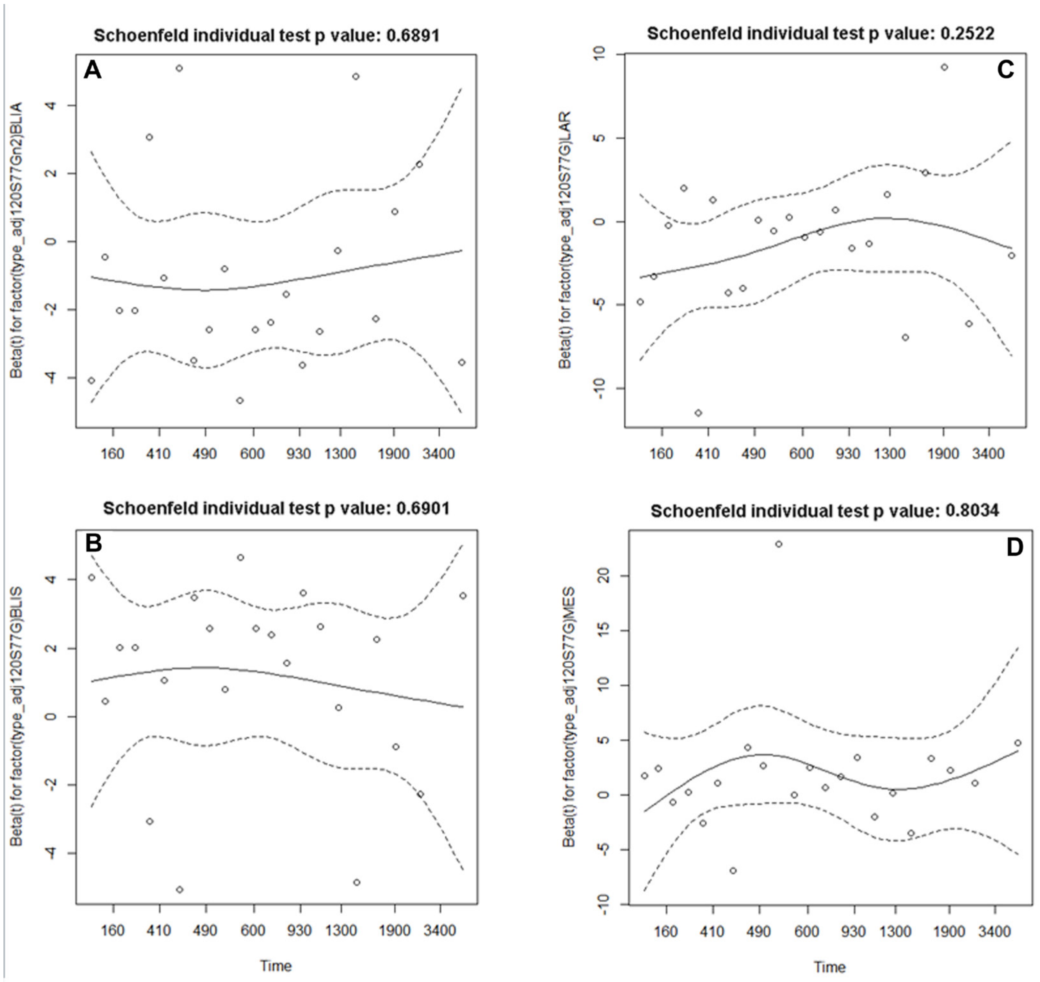

Supplementary Figure 5: Graphs of the scaled Schoenfeld residuals against the transformed time for the primary predictor (subtypes with four levels). (**A**) Subtype BLIA; (**B**) Subtype BLIS; (**C**) Subtype LAR; (**D**) Subtype MES.

Supplementary Table 1: 77-gene centroid signatures for four subtypes of TNBC. See_Supplementary_Table_1

Supplementary Table 2: Subtype assignment for 120 samples. See_Supplementary_Table_2
