# Supplementary material for: Molecular subtypes of triple-negative breast cancer in women of different race and ethnicity
Source: Oncotarget. 2019 Jan 4;10(2):198–208. doi: 10.18632/oncotarget.26559 (PMC6349443; doi:10.18632/oncotarget.26559)
Supplement: Supplementary file 2 [file oncotarget-10-198-s002.docx]

Supplemental Table 1. 77-gene centroid signatures for four subtypes of TNBC

| N | Gene | LAR | MES | BLIA | BLIS |
| --- | --- | --- | --- | --- | --- |
| 1 | ADAMDEC1 | 0.3484 | 0.3431 | 0.6600 | 0.3527 |
| 2 | AGR2 | 0.8574 | 0.3444 | 0.3220 | 0.3151 |
| 3 | AGTR1 | 0.3069 | 0.3870 | 0.1080 | 0.1408 |
| 4 | AIM2 | 0.3963 | 0.4076 | 0.6444 | 0.4182 |
| 5 | AR | 0.8947 | 0.6124 | 0.5004 | 0.5949 |
| 6 | BCL11A | 0.2122 | 0.5166 | 0.7148 | 0.7856 |
| 7 | BST2 | 0.9191 | 0.8331 | 0.9063 | 0.7856 |
| 8 | CA12 | 0.8282 | 0.5532 | 0.5090 | 0.5372 |
| 9 | CCL5 | 0.6505 | 0.6729 | 0.8306 | 0.6633 |
| 10 | CD2 | 0.6686 | 0.6463 | 0.8331 | 0.5990 |
| 11 | CD36 | 0.4819 | 0.7460 | 0.3670 | 0.3903 |
| 12 | CDH1 | 0.8261 | 0.5751 | 0.6735 | 0.7971 |
| 13 | CFD | 0.8309 | 0.9269 | 0.6854 | 0.6350 |
| 14 | CHI3L1 | 0.4388 | 0.7168 | 0.8003 | 0.8351 |
| 15 | COL9A3 | 0.4644 | 0.4076 | 0.5033 | 0.7545 |
| 16 | CXCL11 | 0.2995 | 0.1875 | 0.6890 | 0.3494 |
| 17 | CXCL9 | 0.8457 | 0.7387 | 0.9709 | 0.7831 |
| 18 | DHRS2 | 0.6133 | 0.1297 | 0.1338 | 0.1432 |
| 19 | DPT | 0.7128 | 0.8457 | 0.5925 | 0.6125 |
| 20 | DSC2 | 0.4298 | 0.4016 | 0.6436 | 0.7692 |
| 21 | EDNRB | 0.2681 | 0.5977 | 0.2238 | 0.2463 |
| 22 | ENPP2 | 0.5963 | 0.8444 | 0.6207 | 0.4877 |
| 23 | EPHB3 | 0.5399 | 0.5259 | 0.5949 | 0.7889 |
| 24 | ERBB4 | 0.3085 | 0.1057 | 0.0884 | 0.0966 |
| 25 | EZH2 | 0.6537 | 0.3783 | 0.8547 | 0.8106 |
| 26 | FGL2 | 0.6569 | 0.7580 | 0.7443 | 0.5295 |
| 27 | FHL1 | 0.3915 | 0.6184 | 0.2897 | 0.3441 |
| 28 | FOXA1 | 0.6468 | 0.0691 | 0.0978 | 0.0655 |
| 29 | FOXC1 | 0.6468 | 0.8338 | 0.9063 | 0.9689 |
| 30 | GABRP | 0.2505 | 0.6742 | 0.8453 | 0.9787 |
| 31 | GBP1 | 0.7340 | 0.7181 | 0.9165 | 0.7602 |
| 32 | GBP5 | 0.4197 | 0.4069 | 0.7193 | 0.4276 |
| 33 | GPX3 | 0.7734 | 0.8976 | 0.6854 | 0.7402 |
| 34 | GZMB | 0.3372 | 0.4754 | 0.7565 | 0.3670 |
| 35 | HBB | 0.7559 | 0.8737 | 0.6330 | 0.7021 |
| 36 | HERC5 | 0.6117 | 0.6210 | 0.8478 | 0.7136 |
| 37 | HLA.DMA | 0.8596 | 0.8910 | 0.9051 | 0.8065 |
| 38 | IGF1 | 0.6085 | 0.7926 | 0.4832 | 0.5274 |
| 39 | IGSF6 | 0.3452 | 0.3318 | 0.4051 | 0.2770 |
| 40 | IRX1 | 0.4090 | 0.5951 | 0.5458 | 0.8498 |
| 41 | KCNK5 | 0.4665 | 0.4927 | 0.6678 | 0.8044 |
| 42 | LAMP3 | 0.5213 | 0.5439 | 0.8924 | 0.7766 |
| 43 | LEP | 0.3367 | 0.6004 | 0.2791 | 0.3081 |
| 44 | LPL | 0.6723 | 0.9176 | 0.6727 | 0.7868 |
| 45 | MEOX2 | 0.0207 | 0.0525 | 0.0209 | 0.0176 |
| 46 | MIA | 0.5005 | 0.6210 | 0.6911 | 0.8748 |
| 47 | NAT1 | 0.9085 | 0.5844 | 0.5094 | 0.5061 |
| 48 | OGN | 0.4441 | 0.6576 | 0.0994 | 0.1244 |
| 49 | PBK | 0.5255 | 0.3198 | 0.7946 | 0.7021 |
| 50 | PIP | 0.9213 | 0.6070 | 0.5880 | 0.6363 |
| 51 | PLAT | 0.8574 | 0.7387 | 0.5020 | 0.7181 |
| 52 | PROM1 | 0.3043 | 0.6257 | 0.9014 | 0.9210 |
| 53 | PSAT1 | 0.4239 | 0.5971 | 0.7541 | 0.7332 |
| 54 | PSMB9 | 0.9085 | 0.9089 | 0.9738 | 0.8895 |
| 55 | PTGER4 | 0.5160 | 0.5838 | 0.5491 | 0.4574 |
| 56 | RARRES3 | 0.8170 | 0.6729 | 0.7578 | 0.5777 |
| 57 | RHOH | 0.4606 | 0.2640 | 0.3580 | 0.2099 |
| 58 | SCARA5 | 0.2191 | 0.4973 | 0.2345 | 0.2402 |
| 59 | SCUBE2 | 0.8101 | 0.5372 | 0.2664 | 0.3515 |
| 60 | SEMA5A | 0.7170 | 0.6961 | 0.5078 | 0.6232 |
| 61 | SERPINB5 | 0.3128 | 0.3025 | 0.4006 | 0.5483 |
| 62 | SFRP1 | 0.4176 | 0.8271 | 0.7966 | 0.9030 |
| 63 | SIDT1 | 0.7122 | 0.1350 | 0.1739 | 0.0777 |
| 64 | SLAMF7 | 0.3468 | 0.3783 | 0.5160 | 0.3707 |
| 65 | SOX10 | 0.5351 | 0.5459 | 0.5818 | 0.7508 |
| 66 | SPOCK1 | 0.8590 | 0.7906 | 0.5634 | 0.7799 |
| 67 | SPON1 | 0.5793 | 0.7021 | 0.3768 | 0.5872 |
| 68 | SRPX | 0.7660 | 0.9402 | 0.7001 | 0.7917 |
| 69 | STAT1 | 0.6798 | 0.5838 | 0.7807 | 0.6633 |
| 70 | TAP1 | 0.8574 | 0.8245 | 0.9570 | 0.8752 |
| 71 | TAP2 | 0.4606 | 0.4521 | 0.6256 | 0.5327 |
| 72 | TFF1 | 0.8303 | 0.4761 | 0.4034 | 0.4525 |
| 73 | TFF3 | 0.8505 | 0.4129 | 0.3539 | 0.3613 |
| 74 | TIMP4 | 0.3011 | 0.5938 | 0.2594 | 0.2602 |
| 75 | TSHZ3 | 0.6771 | 0.6503 | 0.5061 | 0.5700 |
| 76 | TTYH1 | 0.1479 | 0.2507 | 0.3081 | 0.5696 |
| 77 | ZBTB16 | 0.3888 | 0.4295 | 0.1780 | 0.1710 |
