# Supplementary material for: Molecular subtypes of triple-negative breast cancer in women of different race and ethnicity
Source: Oncotarget. 2019 Jan 4;10(2):198–208. doi: 10.18632/oncotarget.26559 (PMC6349443; doi:10.18632/oncotarget.26559)
Supplement: Supplementary file 3 [file oncotarget-10-198-s003.docx]

|  |
| --- |

| Supplemental Table 2. Subtype assignment for the 120 test samples using the Spearman correlation. |
| --- |

| id | Rho_  LAR | Rho_  MES | Rho_  BLIA | Rho_  BLIS | P_  LAR | P_  MES | P_  BLIA | P_  BLIS | Difference.  top2* | Subtype | Subtype_  mixed** | Race ethnicity |
| --- | --- | --- | --- | --- | --- | --- | --- | --- | --- | --- | --- | --- |
| S205.3 | 0.4220 | 0.3854 | 0.7034 | 0.3411 | 0.0002 | 0.0007 | 0.0000 | 0.0030 | 0.2814 | BLIA | BLIA | White,  five control  samples with  Affymetrix  array data |
| S2300.1 | 0.3514 | 0.7074 | 0.7106 | 0.5593 | 0.0022 | 0.0000 | 0.0000 | 0.0000 | 0.0032 | BLIA | BLIA-MES |  |
| S4816.2 | 0.3917 | 0.4934 | 0.5196 | 0.7697 | 0.0006 | 0.0000 | 0.0000 | 0.0000 | 0.2501 | BLIS | BLIS |  |
| S8318.2 | 0.2276 | 0.2722 | 0.3259 | 0.6710 | 0.0515 | 0.0197 | 0.0047 | 0.0000 | 0.345 | BLIS | BLIS |  |
| S9918.2 | 0.5203 | 0.6744 | 0.0294 | 0.1673 | 0.0000 | 0.0000 | 0.8016 | 0.1532 | 0.1541 | MES | MES |  |
| 43065 | 0.3942 | 0.5878 | 0.8384 | 0.5889 | 0.0005 | 0.0000 | 0.0000 | 0.0000 | 0.2495 | BLIA | BLIA | Asian |
| 43066 | 0.4036 | 0.6496 | 0.5192 | 0.7781 | 0.0004 | 0.0000 | 0.0000 | 0.0000 | 0.1285 | BLIS | BLIS | Asian |
| 43753 | 0.3028 | 0.5910 | 0.8705 | 0.7569 | 0.0090 | 0.0000 | 0.0000 | 0.0000 | 0.1135 | BLIA | BLIA | White |
| 43754 | 0.3662 | 0.4404 | 0.8424 | 0.5423 | 0.0014 | 0.0001 | 0.0000 | 0.0000 | 0.3001 | BLIA | BLIA | White |
| 43755 | 0.2442 | 0.4856 | 0.8451 | 0.7053 | 0.0369 | 0.0000 | 0.0000 | 0.0000 | 0.1398 | BLIA | BLIA | White |
| 43756 | 0.4598 | 0.6722 | 0.7425 | 0.6887 | 0.0000 | 0.0000 | 0.0000 | 0.0000 | 0.0538 | BLIA | BLIA_BLIS | White |
| 43758 | 0.7983 | 0.4832 | 0.2465 | 0.2013 | 0.0000 | 0.0000 | 0.0351 | 0.0863 | 0.3151 | LAR | LAR | White |
| 43759 | 0.3559 | 0.2515 | 0.3491 | 0.6140 | 0.0019 | 0.0318 | 0.0024 | 0.0000 | 0.2581 | BLIS | BLIS | White |
| 43760 | 0.4351 | 0.6197 | 0.7941 | 0.7689 | 0.0001 | 0.0000 | 0.0000 | 0.0000 | 0.0252 | BLIA | BLIA_BLIS | White |
| 43761 | 0.3871 | 0.643 | 0.7004 | 0.9154 | 0.0007 | 0.0000 | 0.0000 | 0.0000 | 0.215 | BLIS | BLIS | White |
| 43762 | 0.7760 | 0.4444 | 0.1444 | 0.1940 | 0.0000 | 0.0001 | 0.2186 | 0.0986 | 0.3317 | LAR | LAR | White |
| 43763 | 0.2360 | 0.4892 | 0.7854 | 0.8113 | 0.0438 | 0.0000 | 0.0000 | 0.0000 | 0.0259 | BLIS | BLIA_BLIS | White |
| 43765 | 0.2476 | 0.3275 | 0.7502 | 0.7613 | 0.0345 | 0.0045 | 0.0000 | 0.0000 | 0.0112 | BLIS | BLIA_BLIS | White |
| 43766 | 0.4812 | 0.5694 | 0.7061 | 0.4119 | 0.0000 | 0.0000 | 0.0000 | 0.0003 | 0.1366 | BLIA | BLIA | White |
| 43767 | 0.8413 | 0.4596 | 0.1901 | 0.1841 | 0.0000 | 0.0000 | 0.1054 | 0.1167 | 0.3817 | LAR | LAR | White |
| 43768 | 0.8078 | 0.5176 | 0.4779 | 0.3476 | 0.0000 | 0.0000 | 0.0000 | 0.0025 | 0.2902 | LAR | LAR | Asian |
| 43769 | 0.6475 | 0.6441 | 0.4308 | 0.4518 | 0.0000 | 0.0000 | 0.0001 | 0.0001 | 0.0034 | LAR | LAR | White |
| 43770 | 0.8512 | 0.4751 | 0.2476 | 0.1947 | 0.0000 | 0.0000 | 0.0345 | 0.0975 | 0.3761 | LAR | LAR | White |
| 43771 | 0.3197 | 0.5736 | 0.8232 | 0.5485 | 0.0057 | 0.0000 | 0.0000 | 0.0000 | 0.2497 | BLIA | BLIA | White |
| 43772 | 0.2474 | 0.6920 | 0.7242 | 0.7790 | 0.0345 | 0.0000 | 0.0000 | 0.0000 | 0.0548 | BLIS | BLIA_BLIS | African American |
| 43773 | 0.8072 | 0.3126 | 0.3303 | 0.1850 | 0.0000 | 0.0069 | 0.0042 | 0.1151 | 0.4769 | LAR | LAR | White |
| 43774 | 0.4415 | 0.5700 | 0.7455 | 0.6330 | 0.0001 | 0.0000 | 0.0000 | 0.0000 | 0.1125 | BLIA | BLIA | African American |
| 43775 | 0.3183 | 0.5900 | 0.6838 | 0.8294 | 0.0059 | 0.0000 | 0.0000 | 0.0000 | 0.1456 | BLIS | BLIS | Asian |
| 43776 | 0.7033 | 0.3128 | -0.0448 | 0.0706 | 0.0000 | 0.0069 | 0.7033 | 0.5487 | 0.3906 | LAR | LAR | Asian |
| 43777 | 0.1961 | 0.4078 | 0.8626 | 0.8237 | 0.0952 | 0.0003 | 0.0000 | 0.0000 | 0.0389 | BLIA | BLIA_BLIS | Asian |
| 43778 | 0.8839 | 0.4907 | 0.1504 | 0.1660 | 0.0000 | 0.0000 | 0.1996 | 0.1561 | 0.3932 | LAR | LAR | White |
| 43779 | 0.8145 | 0.5152 | 0.1208 | 0.1035 | 0.0000 | 0.0000 | 0.3027 | 0.3782 | 0.2993 | LAR | LAR | African American |
| 43780 | 0.2499 | 0.4232 | 0.6822 | 0.8421 | 0.0328 | 0.0002 | 0.0000 | 0.0000 | 0.1599 | BLIS | BLIS | White |
| 43781 | 0.3747 | 0.6832 | 0.8240 | 0.8095 | 0.0011 | 0.0000 | 0.0000 | 0.0000 | 0.0145 | BLIA | BLIA_BLIS | Hispanic |
| 43782 | 0.2311 | 0.3951 | 0.8222 | 0.8576 | 0.0480 | 0.0005 | 0.0000 | 0.0000 | 0.0354 | BLIS | BLIA_BLIS | White |
| 43783 | 0.1924 | 0.5414 | 0.3929 | 0.7996 | 0.1013 | 0.0000 | 0.0006 | 0.0000 | 0.2582 | BLIS | BLIS | White |
| 43784 | 0.7256 | 0.2340 | -0.0514 | 0.1061 | 0.0000 | 0.0453 | 0.6628 | 0.3667 | 0.4916 | LAR | LAR | Asian |
| 43785 | 0.3323 | 0.6374 | 0.7589 | 0.8917 | 0.0039 | 0.0000 | 0.0000 | 0.0000 | 0.1327 | BLIS | BLIS | White |
| 43786 | 0.1768 | 0.3898 | 0.9250 | 0.7299 | 0.1314 | 0.0006 | 0.0000 | 0.0000 | 0.1951 | BLIA | BLIA | White |
| 43787 | 0.4564 | 0.7033 | 0.6827 | 0.6700 | 0.0000 | 0.0000 | 0.0000 | 0.0000 | 0.0206 | MES | BLIA_BLIS | White |
| 43788 | 0.3869 | 0.6995 | 0.7266 | 0.8087 | 0.0007 | 0.0000 | 0.0000 | 0.0000 | 0.0821 | BLIS | BLIA_BLIS | White |
| 43789 | 0.1574 | 0.4340 | 0.8916 | 0.8404 | 0.1795 | 0.0001 | 0.0000 | 0.0000 | 0.0513 | BLIA | BLIA_BLIS | White |
| 43790 | 0.1796 | 0.5079 | 0.6265 | 0.8768 | 0.1256 | 0.0000 | 0.0000 | 0.0000 | 0.2503 | BLIS | BLIS | White |
| 43791 | 0.1738 | 0.5257 | 0.7677 | 0.8364 | 0.1379 | 0.0000 | 0.0000 | 0.0000 | 0.0686 | BLIS | BLIA_BLIS | White |
| 43792 | 0.2197 | 0.3544 | 0.7114 | 0.8572 | 0.0605 | 0.0020 | 0.0000 | 0.0000 | 0.1458 | BLIS | BLIS | White |
| 43793 | 0.7837 | 0.3652 | 0.4073 | 0.1839 | 0.0000 | 0.0014 | 0.0003 | 0.1170 | 0.3764 | LAR | LAR | Asian |
| 43795 | 0.7034 | 0.4623 | 0.4155 | 0.3373 | 0.0000 | 0.0000 | 0.0003 | 0.0034 | 0.2412 | LAR | LAR | White |
| 43796 | 0.4578 | 0.7511 | 0.3040 | 0.3387 | 0.0000 | 0.0000 | 0.0087 | 0.0033 | 0.2933 | MES | MES | White |
| 43797 | 0.2359 | 0.4281 | 0.8529 | 0.8454 | 0.0438 | 0.0002 | 0.0000 | 0.0000 | 0.0076 | BLIA | BLIA_BLIS | Hispanic |
| 43798 | 0.6278 | 0.5079 | 0.3856 | 0.4150 | 0.0000 | 0.0000 | 0.0007 | 0.0003 | 0.1199 | LAR | LAR | White |
| 43799 | 0.2635 | 0.5259 | 0.8214 | 0.7822 | 0.0242 | 0.0000 | 0.0000 | 0.0000 | 0.0392 | BLIA | BLIA_BLIS | African American |
| 43800 | 0.7810 | 0.3897 | 0.0224 | 0.0825 | 0.0000 | 0.0006 | 0.8466 | 0.4839 | 0.3913 | LAR | LAR | Hispanic |
| 43802 | 0.2767 | 0.4921 | 0.8348 | 0.8247 | 0.0177 | 0.0000 | 0.0000 | 0.0000 | 0.0101 | BLIA | BLIA_BLIS | Hispanic |
| 43803 | 0.3607 | 0.6439 | 0.7427 | 0.8737 | 0.0017 | 0.0000 | 0.0000 | 0.0000 | 0.131 | BLIS | BLIS | Hispanic |
| 43804 | 0.8234 | 0.3887 | 0.1731 | 0.1887 | 0.0000 | 0.0007 | 0.1390 | 0.1079 | 0.4347 | LAR | LAR | Asian |
| 43805 | 0.2792 | 0.3788 | 0.4029 | 0.6399 | 0.0166 | 0.0009 | 0.0004 | 0.0000 | 0.237 | BLIS | BLIS | African American |
| 43806 | 0.3177 | 0.4998 | 0.8927 | 0.7431 | 0.0060 | 0.0000 | 0.0000 | 0.0000 | 0.1495 | BLIA | BLIA | African American |
| 43807 | 0.2348 | 0.4101 | 0.2075 | 0.6106 | 0.0448 | 0.0003 | 0.0769 | 0.0000 | 0.2005 | BLIS | BLIS | African American |
| 43808 | 0.4135 | 0.4795 | 0.8500 | 0.6879 | 0.0003 | 0.0000 | 0.0000 | 0.0000 | 0.1621 | BLIA | BLIA | African American |
| 43809 | 0.3577 | 0.6010 | 0.7104 | 0.8219 | 0.0018 | 0.0000 | 0.0000 | 0.0000 | 0.1114 | BLIS | BLIS | Hispanic |
| 43810 | 0.7709 | 0.6176 | 0.0309 | 0.1343 | 0.0000 | 0.0000 | 0.7929 | 0.2525 | 0.1533 | LAR | LAR | Hispanic |
| 43811 | 0.2660 | 0.4757 | 0.8437 | 0.5293 | 0.0229 | 0.0000 | 0.0000 | 0.0000 | 0.3144 | BLIA | BLIA | Asian |
| 43812 | 0.6740 | 0.6438 | 0.0584 | 0.2239 | 0.0000 | 0.0000 | 0.6203 | 0.0555 | 0.0302 | LAR | LAR | African American |
| 43813 | 0.2614 | 0.4725 | 0.8739 | 0.6682 | 0.0254 | 0.0000 | 0.0000 | 0.0000 | 0.2057 | BLIA | BLIA | Asian |
| 43814 | 0.4258 | 0.5763 | 0.7614 | 0.6957 | 0.0002 | 0.0000 | 0.0000 | 0.0000 | 0.0657 | BLIA | BLIA_BLIS | African American |
| 43815 | 0.3687 | 0.6510 | 0.7031 | 0.8367 | 0.0013 | 0.0000 | 0.0000 | 0.0000 | 0.1336 | BLIS | BLIS | African American |
| 43816 | 0.7703 | 0.4947 | 0.0893 | 0.1293 | 0.0000 | 0.0000 | 0.4482 | 0.2710 | 0.2757 | LAR | LAR | Asian |
| 43817 | 0.3705 | 0.6647 | 0.8840 | 0.7669 | 0.0012 | 0.0000 | 0.0000 | 0.0000 | 0.117 | BLIA | BLIA | Asian |
| 43818 | 0.3796 | 0.5195 | 0.5108 | 0.6905 | 0.0009 | 0.0000 | 0.0000 | 0.0000 | 0.1711 | BLIS | BLIS | Hispanic |
| 43819 | 0.7586 | 0.6211 | 0.4089 | 0.4372 | 0.0000 | 0.0000 | 0.0003 | 0.0001 | 0.1375 | LAR | LAR | Asian |
| 43820 | 0.4611 | 0.7172 | 0.7561 | 0.6481 | 0.0000 | 0.0000 | 0.0000 | 0.0000 | 0.0389 | BLIA | BLIA | Hispanic |
| 43821 | 0.2385 | 0.4228 | 0.8817 | 0.6597 | 0.0415 | 0.0002 | 0.0000 | 0.0000 | 0.222 | BLIA | BLIA | Asian |
| 43822 | 0.1834 | 0.4052 | 0.7620 | 0.9089 | 0.1178 | 0.0004 | 0.0000 | 0.0000 | 0.1469 | BLIS | BLIS | African American |
| 43823 | 0.4847 | 0.6953 | 0.5643 | 0.7386 | 0.0000 | 0.0000 | 0.0000 | 0.0000 | 0.0433 | BLIS | BLIS | Asian |
| 43824 | 0.2925 | 0.5679 | 0.6509 | 0.7723 | 0.0118 | 0.0000 | 0.0000 | 0.0000 | 0.1214 | BLIS | BLIS | Hispanic |
| 43825 | 0.4527 | 0.5135 | 0.8720 | 0.6509 | 0.0001 | 0.0000 | 0.0000 | 0.0000 | 0.221 | BLIA | BLIA | Asian |
| 43826 | 0.8472 | 0.3570 | 0.0814 | 0.1774 | 0.0000 | 0.0019 | 0.4889 | 0.1304 | 0.4902 | LAR | LAR | Asian |
| 43827 | 0.2346 | 0.4404 | 0.5084 | 0.8596 | 0.0449 | 0.0001 | 0.0000 | 0.0000 | 0.3512 | BLIS | BLIS | Asian |
| 43828 | 0.3863 | 0.5104 | 0.8388 | 0.8023 | 0.0007 | 0.0000 | 0.0000 | 0.0000 | 0.0365 | BLIA | BLIA_BLIS | Asian |
| 43829 | 0.2098 | 0.4109 | 0.8404 | 0.7995 | 0.0737 | 0.0003 | 0.0000 | 0.0000 | 0.041 | BLIA | BLIA_BLIS | Asian |
| 43830 | 0.1545 | 0.4982 | 0.7790 | 0.8456 | 0.1874 | 0.0000 | 0.0000 | 0.0000 | 0.0666 | BLIS | BLIA_BLIS | Hispanic |
| 43831 | 0.2981 | 0.3793 | 0.9170 | 0.6472 | 0.0102 | 0.0009 | 0.0000 | 0.0000 | 0.2698 | BLIA | BLIA | White |
| 43832 | 0.4854 | 0.6238 | 0.7286 | 0.4721 | 0.0000 | 0.0000 | 0.0000 | 0.0000 | 0.1047 | BLIA | BLIA | Hispanic |
| 43833 | 0.4195 | 0.6932 | 0.7339 | 0.7502 | 0.0002 | 0.0000 | 0.0000 | 0.0000 | 0.0163 | BLIS | BLIA_BLIS | Hispanic |
| 43834 | 0.6211 | 0.6120 | 0.5324 | 0.3833 | 0.0000 | 0.0000 | 0.0000 | 0.0008 | 0.0091 | LAR | LAR | Hispanic |
| 43835 | 0.4205 | 0.6547 | 0.5377 | 0.7115 | 0.0002 | 0.0000 | 0.0000 | 0.0000 | 0.0568 | BLIS | BLIS | Hispanic |
| 43836 | 0.2904 | 0.6709 | 0.5923 | 0.8582 | 0.0125 | 0.0000 | 0.0000 | 0.0000 | 0.1873 | BLIS | BLIS | Hispanic |
| 43837 | 0.2577 | 0.4693 | 0.6521 | 0.7828 | 0.0276 | 0.0000 | 0.0000 | 0.0000 | 0.1307 | BLIS | BLIS | Hispanic |
| 43838 | 0.3625 | 0.6151 | 0.8550 | 0.6408 | 0.0016 | 0.0000 | 0.0000 | 0.0000 | 0.2142 | BLIA | BLIA | Hispanic |
| 43839 | 0.1233 | 0.6951 | 0.4044 | 0.6383 | 0.2931 | 0.0000 | 0.0004 | 0.0000 | 0.0568 | MES | BLIS | Hispanic |
| 43840 | 0.2408 | 0.4451 | 0.5120 | 0.8250 | 0.0396 | 0.0001 | 0.0000 | 0.0000 | 0.313 | BLIS | BLIS | Hispanic |
| 43841 | 0.2650 | 0.4598 | 0.8964 | 0.6649 | 0.0234 | 0.0000 | 0.0000 | 0.0000 | 0.2315 | BLIA | BLIA | Hispanic |
| 43842 | 0.3778 | 0.6040 | 0.8111 | 0.9231 | 0.0009 | 0.0000 | 0.0000 | 0.0000 | 0.112 | BLIS | BLIS | Hispanic |
| 43843 | 0.3595 | 0.6535 | 0.6123 | 0.6479 | 0.0017 | 0.0000 | 0.0000 | 0.0000 | 0.0056 | MES | BLIA_BLIS | Hispanic |
| 43844 | 0.4405 | 0.7176 | 0.6488 | 0.8739 | 0.0001 | 0.0000 | 0.0000 | 0.0000 | 0.1563 | BLIS | BLIS | Hispanic |
| 43845 | 0.6483 | 0.4283 | 0.5257 | 0.2282 | 0.0000 | 0.0002 | 0.0000 | 0.0509 | 0.1226 | LAR | LAR | Hispanic |
| 43846 | 0.3495 | 0.6521 | 0.5485 | 0.8282 | 0.0023 | 0.0000 | 0.0000 | 0.0000 | 0.1761 | BLIS | BLIS | Hispanic |
| 43847 | 0.2463 | 0.5077 | 0.9174 | 0.7625 | 0.0352 | 0.0000 | 0.0000 | 0.0000 | 0.1549 | BLIA | BLIA | Hispanic |
| 43848 | 0.3028 | 0.5001 | 0.8935 | 0.7662 | 0.0090 | 0.0000 | 0.0000 | 0.0000 | 0.1274 | BLIA | BLIA | Hispanic |
| 43849 | 0.3537 | 0.6803 | 0.7283 | 0.8299 | 0.0021 | 0.0000 | 0.0000 | 0.0000 | 0.1015 | BLIS | BLIS | Hispanic |
| 43882 | 0.3514 | 0.5899 | 0.6631 | 0.6686 | 0.0022 | 0.0000 | 0.0000 | 0.0000 | 0.0054 | BLIS | BLIA_BLIS | Hispanic |
| 43883 | 0.1390 | 0.3926 | 0.7759 | 0.8563 | 0.2363 | 0.0006 | 0.0000 | 0.0000 | 0.0804 | BLIS | BLIA_BLIS | Hispanic |
| 43884 | 0.2321 | 0.2951 | 0.8306 | 0.6589 | 0.0472 | 0.0111 | 0.0000 | 0.0000 | 0.1718 | BLIA | BLIA | Hispanic |
| 43885 | 0.2308 | 0.3836 | 0.8894 | 0.7158 | 0.0483 | 0.0008 | 0.0000 | 0.0000 | 0.1736 | BLIA | BLIA | Hispanic |
| 43887 | 0.3671 | 0.3701 | 0.7744 | 0.6461 | 0.0014 | 0.0012 | 0.0000 | 0.0000 | 0.1283 | BLIA | BLIA | Hispanic |
| 43888 | 0.3099 | 0.6069 | 0.7818 | 0.8241 | 0.0074 | 0.0000 | 0.0000 | 0.0000 | 0.0423 | BLIS | BLIA_BLIS | Hispanic |
| 43889 | 0.2406 | 0.4133 | 0.9437 | 0.7048 | 0.0398 | 0.0003 | 0.0000 | 0.0000 | 0.2389 | BLIA | BLIA | Hispanic |
| 43890 | 0.7501 | 0.5432 | 0.3968 | 0.2551 | 0.0000 | 0.0000 | 0.0005 | 0.0293 | 0.2069 | LAR | LAR | Hispanic |
| 43891 | 0.2551 | 0.4009 | 0.7758 | 0.8613 | 0.0293 | 0.0004 | 0.0000 | 0.0000 | 0.0855 | BLIS | BLIA_BLIS | Hispanic |
| 43892 | 0.2838 | 0.6966 | 0.4493 | 0.802 | 0.0148 | 0.0000 | 0.0001 | 0.0000 | 0.1053 | BLIS | BLIS | Hispanic |
| 43893 | 0.4269 | 0.6910 | 0.7444 | 0.7137 | 0.0002 | 0.0000 | 0.0000 | 0.0000 | 0.0307 | BLIA | BLIA_BLIS | Hispanic |
| 43894 | 0.3502 | 0.7239 | 0.6800 | 0.7972 | 0.0023 | 0.0000 | 0.0000 | 0.0000 | 0.0732 | BLIS | BLIS | Hispanic |
| 43895 | 0.5352 | 0.6630 | 0.4147 | 0.7215 | 0.0000 | 0.0000 | 0.0003 | 0.0000 | 0.0585 | BLIS | BLIS | Hispanic |
| 43896 | 0.7872 | 0.6123 | 0.2026 | 0.1756 | 0.0000 | 0.0000 | 0.0845 | 0.1339 | 0.1749 | LAR | LAR | Hispanic |
| 43897 | 0.4389 | 0.7134 | 0.7515 | 0.7696 | 0.0001 | 0.0000 | 0.0000 | 0.0000 | 0.0181 | BLIS | BLIA_BLIS | Hispanic |
| 43898 | 0.3452 | 0.6215 | 0.7406 | 0.8831 | 0.0027 | 0.0000 | 0.0000 | 0.0000 | 0.1425 | BLIS | BLIS | Hispanic |
| 43899 | 0.4198 | 0.6868 | 0.7053 | 0.8038 | 0.0002 | 0.0000 | 0.0000 | 0.0000 | 0.0985 | BLIS | BLIA_BLIS | Hispanic |
| 43900 | 0.2829 | 0.5283 | 0.7578 | 0.8450 | 0.0151 | 0.0000 | 0.0000 | 0.0000 | 0.0872 | BLIS | BLIA_BLIS | Hispanic |

*Rho, Spearman Correlation coefficients; P, P values; difference.top2, difference in the Spearman correlation values between the top two subtypes;

** a sample is allowed to be associated with multiple subtypes if the difference.top2 is less than 0.1, but MES is not considered as one of mixed subtypes since MES subtype largely reflects the mixture of stromal or immune cells (non-tumor cells) with epithelial cells; highlighted five samples are control samples with Affymetrix array data.
